# Clinical and Histopathological Aspects of MRONJ in Cancer Patients

**DOI:** 10.3390/jcm12103383

**Published:** 2023-05-10

**Authors:** George Adrian Ciobanu, Laurențiu Mogoantă, Adrian Camen, Mihaela Ionescu, Daniel Vlad, Ionela Elisabeta Staicu, Cristina Maria Munteanu, Mircea Ionuț Gheorghiță, Răzvan Mercuț, Elena Claudia Sin, Sanda Mihaela Popescu

**Affiliations:** 1 Department of Oral Rehabilitation, University of Medicine and Pharmacy of Craiova, 200349 Craiova, Romania; 2 Department of Oral and Maxillofacial Surgery, Dental Medicine Faculty, “Ovidius” University of Constanța, 900470 Constanța, Romania; 3 Department of Histology, University of Medicine and Pharmacy of Craiova, 200349 Craiova, Romania; 4 Department of Oral and Maxillofacial Surgery, University of Medicine and Pharmacy of Craiova, 200349 Craiova, Romania; 5 Department of Medical Informatics and Biostatistics, University of Medicine and Pharmacy of Craiova, 200349 Craiova, Romania; 6 Department of Orthodontics, University of Medicine and Pharmacy of Craiova, 200349 Craiova, Romania; 7 Department of Plastic Surgery, University of Medicine and Pharmacy of Craiova, 200349 Craiova, Romania

**Keywords:** osteonecrosis, zoledronic acid, histopathology, cancer patients, bisphosphonate

## Abstract

Medication-related osteonecrosis of the jaw (MRONJ) is a major complication of bisphosphonate treatment in cancer patients, and its etiology is not completely clarified. The study’s goal is to find connections between the clinical and histopathological characteristics of osteonecrosis and bisphosphonates in a cohort of cancer patients who had osteonecrosis treated surgically. The retrospective study includes 51 patients of both sexes, aged 46 to 85 years, who underwent surgical treatment for MRONJ in two oral and maxillofacial surgery clinics (Craiova and Constanța). Demographic, clinical, and imaging data from the records of patients with osteonecrosis were analyzed. The surgical treatment removed the necrotic bone, and the harvested fragments were analyzed from a histopathological perspective. The histopathological examination data were evaluated and statistically processed to look for viable bone, granulation tissue, bacterial colonies, and inflammatory infiltrate. In the study groups, MRONJ was found particularly in the posterior regions of the mandible. Tooth extraction, but also periapical or periodontal infections, represented the trigger factors in most of the cases. The surgical therapy consisted of sequestrectomy or bone resection, and the histopathological examination of the fragments revealed osteonecrosis-specific features, such as the lack of bone cells, the development of an inflammatory infiltrate, and the existence of bacterial colonies. MRONJ in cancer patients receiving zoledronic acid is a severe complication that significantly lowers quality of life. Since these patients are not usually monitored by the dentist, they are identified in advanced stages of MRONJ. For these patients, thorough dental monitoring could reduce the incidence of osteonecrosis and its related complications.

## 1. Introduction

Breast and prostate cancers, as well as multiple myeloma, lung cancer, and ovarian and digestive cancers, are frequently associated with bone metastases [1,2]. The treatment of choice for these complications consists of antiresorptive and antiangiogenic medicines, such as zoledronic acid, an injectable bisphosphonate [3,4]. Zoledronic acid therapy in cancer bone metastases can last for several years, and the medicine is usually given monthly at a dose of 4 mg per month, in combination with a corticosteroid [5,6].

An adverse effect of bisphosphonate therapy is osteonecrosis of the jaw [7,8], which can be triggered most frequently by oral surgery, such as extractions [9,10,11], but also by inflammatory oral conditions, such as periodontal disease [9,11] and periapical complications of dental caries [9].

The first physician to report the occurrence of osteonecrosis was Robert E. Marx, who published in 2003 the first article regarding this adverse effect occurring in bone metastases from cancers treated with bisphosphonates [12].

The definition of osteonecrosis was later established by the American Association of Oral and Maxillofacial Surgeons (AAOMS) in 2007 [13] and designated it as bisphosphonate-related osteonecrosis of the jaw. In 2014, the American Association of Oral and Maxillofacial Surgeons changed the name to medication-related osteonecrosis of the jaw (MRONJ) since this adverse effect has also been observed with other types of antiresorptive drugs (receptor activator of nuclear factor kappa B ligand (RANKL) inhibitors) and antiangiogenic therapies, not only with bisphosphonates [14].

Osteonecrosis of the jaw associated with bisphosphonates, such as zoledronic acid, occurs in cancer patients in variable percentages depending on the duration of treatment (0.4% up to 1.6% after 1 year of therapy; 0.8% up to 2.1% after 2 years; and 1.0% up to 2.3% after 3 years of administration [15]) and has an impact on patients’ quality of life [16].

Risk factors for MRONJ are represented by drug-related factors [16,17], especially directions of use [16], cumulative dose [15,16], and local factors, such as oral hygiene and oral health status [17,18], oral surgery [19], demographic, systemic, and genetic factors [19,20,21].

The aim of the study was to correlate demographic, clinical, imaging, and histopathological aspects of MRONJ in cancer patients surgically treated for MRONJ in two oral and maxillofacial surgery clinics from Craiova and Constanța.

## 2. Materials and Methods

The retrospective study analyzed archived materials collected between October 2017 and June 2022. The record files of 51 patients diagnosed and treated for MRONJ with zoledronic acid in the Clinics of Oral and Maxillofacial (OMF) Surgery in Craiova and Constanța were analyzed.

The ethics committee of the University of Medicine and Pharmacy of Craiova approved this study by decision no. 59/22.03.2019. All patients provided their informed consent regarding the use of their personal and clinical data for education and research purposes.

### 2.1. Study Inclusion Criteria

All patients included in the current study were treated with intravenous (IV) zoledronic acid for bone metastases and were later diagnosed and surgically treated for MRONJ. The surgical treatment was carried out by curettage and sequestrectomy in most cases, with beveling of the sharp bone edges and covering of the wound with a muco-periosteal flap, and in relapsed cases, a second surgical intervention included bone resection and osteosynthesis and reconstruction with proximity flaps. In all cases, the removal of bone sequestrations and curettage in the bone tissue were performed until clear bleeding appeared from the underlying bone. A-PRF was used in very few cases, especially when the resulting bone defect could not be covered with muco-periosteal flap, or when it was difficult to protect the remaining bone.

Due to the long half-life of zoledronic acid, the surgeon did not recommend stopping its administration. The treatment with zoledronic acid was stopped only in patients that received this recommendation from their oncologists.

As recommended by AAOMS in 2022 [8], the diagnostic criteria for MRONJ were the following: (i) current or previous treatment based on antiresorptive or antiangiogenic agents; (ii) the presence of exposed bone or intraoral or extraoral fistula in the maxillofacial region that has lasted for more than eight weeks; and (iii) patients with no history of radiotherapy to the jaw or obvious metastatic disease of the jaw.

The patients with cancers in the jaw area and patients with radiation therapy in the jaw area in the past were excluded from the study.

For each patient, the following data were acquired: gender, age, MRONJ localization, the results of microbiological exams, and the results of the histopathology reports, with the presence of inflammatory infiltrate, lymphoplasmacytic infiltrate, presence of macrophages, and viable bone.

According to the medical charts, after clinical and imagistic confirmation of a MRONJ diagnosis and its stage, patients were treated according to the Guide for MRONJ Treatment issued by Romanian College of Dentists [22] with empirical antibiotherapy. Each patient received local decontamination of the mouth with chlorhexidine 0.12% mouthwash two times a day, topical application on the wound with chlorhexidine gel 1% three times a day, and empirical oral antibiotherapy with amoxicillin and clavulanic acid 875/125 mg two times a day for patients without a beta-lactam allergy, and clindamycin 600 mg two times daily for allergic patients. After surgery, antibiotherapy continued for 10 to 14 days, intravenously, until sutures were removed (in severe cases), followed by oral antibiotherapy for 7 days. In less severe cases, after discharge, the patient continued oral antibiotherapy for up to 2–3 weeks after the surgical procedure.

### 2.2. Microbiological Examination

According to the medical charts, the microbiological examination was performed for each case according to the protocol of antibiogram. Before beginning the surgical intervention, the swab technique was used for all patients to collect samples from purulent discharge using tubes with transport media—swab specimen collection (Deltalab, Amiens Viscosa, Spain). The samples subsequently underwent microbiological examination using routine culture methods in hospital laboratories from the Emergency Clinical Craiova County Hospital in Craiova and Emergency Clinical County Hospital from Constanta. The collected samples were homogenized and streaked on Columbia blood agar (Biomerieux, Marcy L’Étoile, France) and incubated at 37 °C aerobic in the presence of 5% CO_2_ and anaerobic. For 14 days, each plate was read every 48 h. Growing bacteria were differentiated to the species level through biochemical characteristics identification and were subjected to susceptibility testing using a Vitek II system (Biomerieux, Marcy L´Étoile, France). Species identification was used for deducing susceptibility of the species from the resident flora of the oral cavity.

### 2.3. Histological Examination

According to the medical charts, for each case, the histological exam was performed as per the following protocol: the specimens collected after the surgical treatment were fixed in 10% neutral buffered formalin (*v*/*v*) and sent to the Anatomic-pathology Service of the Constanța or Craiova County Clinical Hospital, being processed using the routine histological technique in order to obtain paraffin blocks. For all cases, 3 μm-thick sections were cut on a Leica RM2245 semi-automatic rotary microtome and mounted on histological slides. For the morphological diagnosis, the slides were stained using the hematoxylin-eosin (HE) and the trichrome technique, according to the Goldner–Szekely (GS) method. Immunomarkers used for the cells’ identification were CD3, CD20, and CD68. Histological and immunohistochemical evaluation was performed with a Nikon Eclipse 55i optical microscope (Tokyo, Japan) in the Research Center for Microscopic Morphology and Immunology, University of Medicine and Pharmacy of Craiova.

### 2.4. Statistical Analysis

Microsoft Excel was used to regroup patient data, to convert inputs into categorical parameters, and to perform the descriptive analysis upon the acquired values. Associations between variables and results comparisons were evaluated based on Chi-square tests or Fisher’s exact tests (for inadequate sample sizes for the Chi-square test), using Statistical Package for Social Sciences (SPSS), version 20 (IBM Corp). *p*-value < 0.05 was considered statistically significant.

## 3. Results

**Figure 1:**
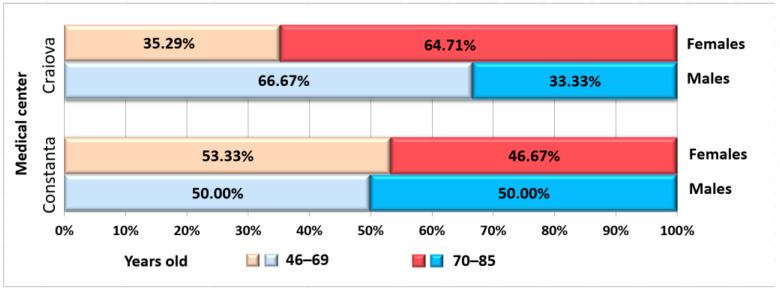
Distribution of patients according to medical center, gender, and age.

The study group included 26 patients from Craiova, and 25 patients from Constanța. Overall, the entire group was imbalanced regarding gender distribution, with 32 females and 19 males. The age of the patients was between 46 and 85 years old (mean age 70.43 ± 8.66 years) (Figure 1).

The types of cancers encountered in MRONJ patients were mostly breast and prostate cancers, both in Craiova and Constanța. Of the 26 patients with MRONJ who came in the oral and maxillo-facial clinic in Craiova, 11 (42.31%) had a breast neoplasm, 7 (26.92%) had a prostate neoplasm, 3 (11.54%) had an ovarian or cervical neoplasm, 2 (7.69%) renal neoplasm, 2 (7.69%) spine cancer, and 1 (3.85%) patient had multiple myeloma. Of the 25 patients who came with MRONJ in the clinic in Constanța, 12 (48%) had breast neoplasm, 8 (32%) had prostate neoplasm, 4 (16%) had colon cancer, and 1 (4%) had lung neoplasm. The patients were treated for cancer with various therapies, especially chemotherapy and hormone therapy. The indication for zoledronic acid administration (4 mg zoledronic acid intravenous solution monthly) was represented by bone metastases in all cases.

All patients came with MRONJ in an advanced stage: stage 2 (37 patients, 72.55%) and stage 3 (14 patients, 27.45%). The trigger factor for MRONJ was represented by extractions in 28 cases (54.91%), periapical infections in 15 cases (29.41%), and periodontal disease in 8 cases (15.68%). From the entire study group, 74.51% were treated surgically by curettage and sequestrectomy and healed afterwards without recurrence; 25.49% relapsed after the first surgical intervention and were treated consequently through surgical resection of the bone, followed by osteosynthesis and primary reconstruction associated with proximity flaps.

**Figure 2:**
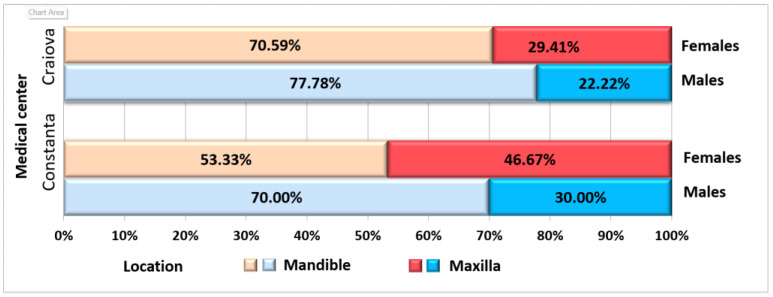
Distribution of patients according to medical center, gender, and MRONJ location.

MRONJ was localized mostly at the mandibular level (66.67% from the entire study group) compared to the maxillary level (33.33%). The group distribution is displayed in Figure 2. The analysis of gender distribution emphasized the following proportions: 62.5% of females presented MRONJ at the mandibular level compared to 73.7% in males. However, no statistically significant association between these two parameters was determined.

From the entire study group, only three patients (5.88%) presented anterior MRONJ (two females and one male), all older than 72 years old. The other 48 patients (94.12%) were diagnosed with posterior MRONJ. Further statistical analysis identified no significant association with the other variables acquired for this study.

Demographic and clinical data are displayed in Table 1.

**Figure 3:**
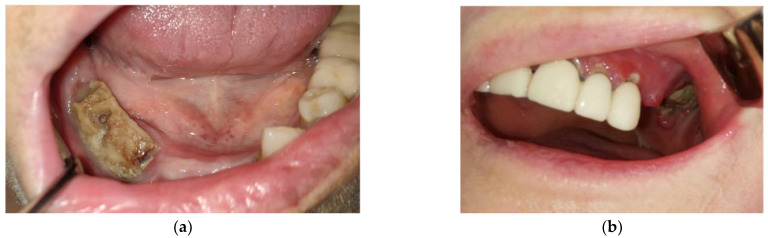
(**a**) Intraoral aspect that highlights the exposure of the alveolar mandibular bone that extends from the canine region to the region of the first molar on the right side. The denuded bone has a grey appearance covered by yellow-grey deposits with fetid odor and purulent discharge. (**b**) Intraoral aspect that highlights the exposure of the maxillary bone with oro-antral fistula in left upper posterior region of the maxilla. The fistula is associated with denuded bone with a grey appearance and yellow-grey deposits.

The signs and symptoms of the MRONJ in patients who came to the OMF clinic were pain in the jaw bones, erythema of the mucous membrane in the affected area, local purulent discharge, and the presence of exposed bone in the affected mandibular area (Figure 3).

**Figure 4:**
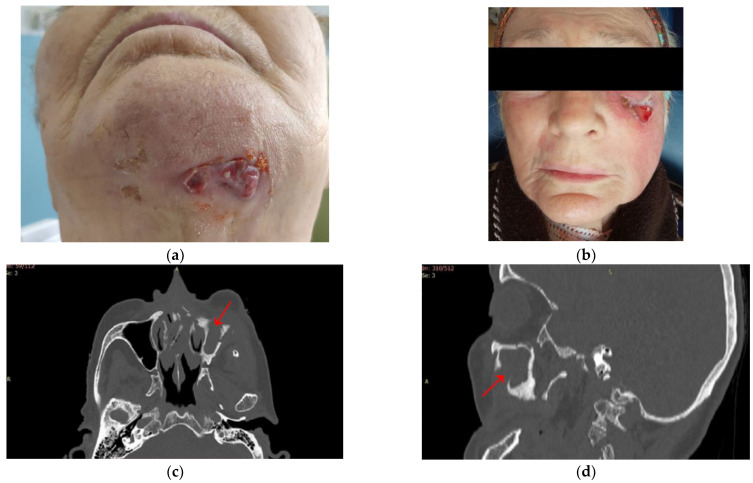
(**a**) Extraoral appearance. Female patient with high bone osteonecrosis in the submental region, with extensive infection. This infection has become evident through a chronic submandibular fistula. (**b**) Extraoral appearance. Female patient with high bone osteonecrosis in the left zygomatic region, with maxillary sinus involvement. This infection has become evident through a chronic facial fistula. (**c**) CT axial section showing opacification of the left maxillary sinus. (**d**) CT sagittal section showing an oro-antral communication on the left side and opacification of the left maxillary sinus.

Patients diagnosed with MRONJ stage 3 had extraoral fistulas (Figure 4) or oro-antral communication.

**Figure 5:**
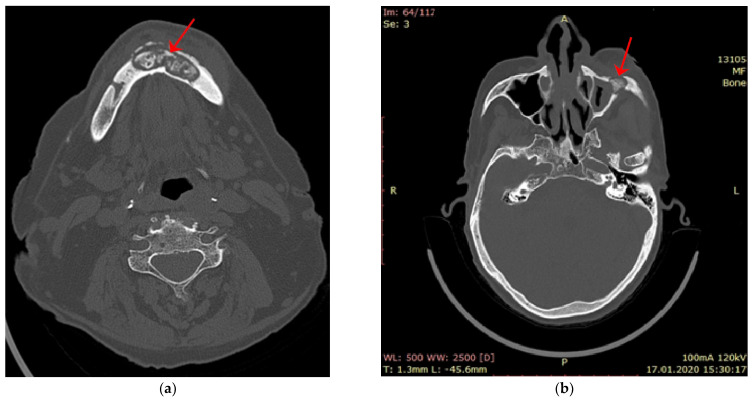
CT image: (**a**) mandibular axial section that shows a bone sequestrum in the mandibular arch region, radio-transparency (osteolysis), which surrounds a radio-opacity area; (**b**) maxillary axial section that shows a sequestrum in the left zygomatic bone, radio-transparency (osteolysis), which surrounds a radio-opacity area.

The patients who came with MRONJ in the surgery clinic were investigated by computer tomograph scanning. For most of them, a CT scan was performed both to clarify the diagnosis and assess the extent of the bone lesion but also to establish the surgical treatment plan. The changes produced at the bone level by MRONJ observed on the tomographic images were the following: areas of bone sequestrum surrounded by a radiolucent area of osteolysis (Figure 5, CT image), opacification of the maxillary sinus, and areas of reactive new bone formation.

Pre-surgical antibiotic treatment consisted of amoxicillin and clavulanic acid in 39 patients (76.47%) and clindamycin in 12 patients (23.53%).

Bacterial colonies were identified during microbiological exams for more than half of the study group (27 patients, representing 52.94%) (Table 2). However, 76.9% of patients from Craiova developed infections, compared to only 28% of patients from Constanța, thus leading to a statistically significant difference between the two cities, χ^2^(1) = 12.244, *p* < 0.0005. There was no significant association with gender, age, the presence of lymphoplasmacytic infiltrate, macrophages, or viable bone (*p* > 0.05).

The predominant bacterial species were *Staphylococcus aureus* and *Escherichia coli*, while *Streptococcus anginosus* and *Pseudomonas mendocina* were identified in smaller percentages. *Streptococcus anginosus* and *Escherichia coli* were susceptible to azithromycin, cefotaxime, ceftriaxone, clindamycin, and vancomycin. The antibiogram showed susceptibility of *Staphylococcus aureus* and *Pseudomonas mendocina* to penicillin, ceftazidime, ceftriaxone, and ciprofloxacin and resistance to clindamycin.

**Figure 6:**
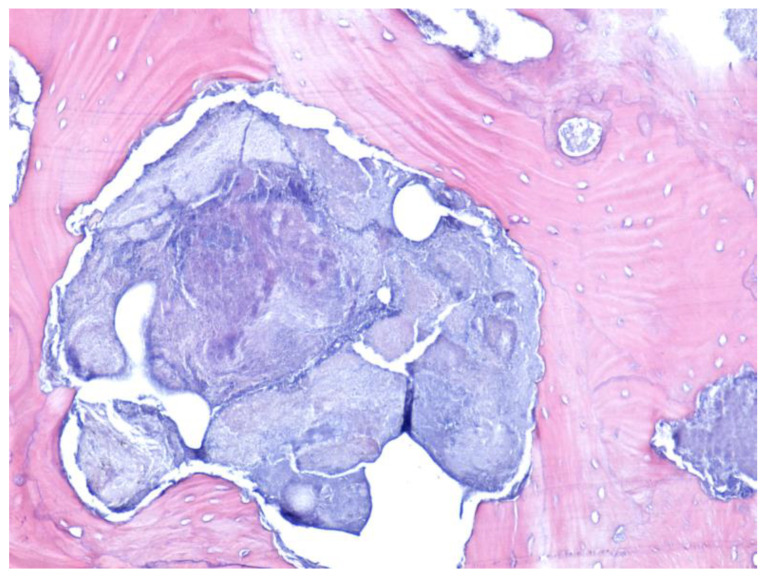
Osteonecrosis in a central bone sequestrum with scalloping border. Multiple outbreaks of osteonecrosis with sharp, scalloping bone borders and empty osteocytic lacunae (devitalized bone) (HE staining, ×100).

**Figure 7:**
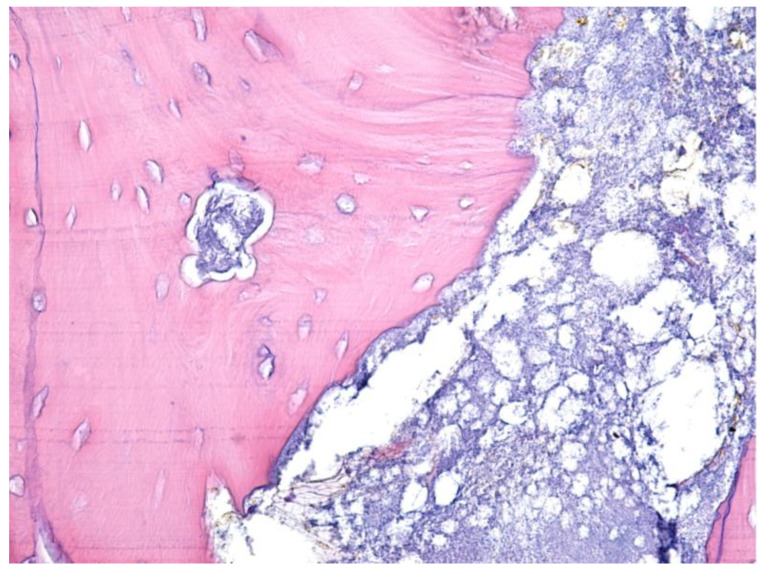
Osteonecrosis with a non-homogeneous aspect and with vacuolar, “moth-eaten” border appearance. Haversian canal with bone necrosis in the middle, empty osteocytic lacunae; non-vital bone with vacuolar aspect and without osteoclasts and osteoblasts (HE staining, ×200).

In all cases included in the study, surgical treatment was performed with sequestrectomy (90.19%) or bone resection (9.81%). The harvested bone and mucosal fragments were sent for histopathological analysis. HE or trichromic staining was performed for the histopathological examination (Figure 6 and Figure 7).

MRONJ-evaluated samples presented areas of bone osteonecrosis with bone tissue in varied moments of dissolution from viable bone tissue, incipient osteonecrosis bone, and bone with complete osteonecrosis. The bone had a mosaic pattern with advanced bone osteonecrosis areas with empty osteocytic lacunae, lack of surrounding immune reaction, and the absence of osteoblasts and osteoclasts. The areas of advanced osteonecrosis were characterized by inhomogeneous appearance, with irregular margins and a vacuolar, moth-eaten appearance. The Haversian canals showed bone necrosis in the center and the osteocyte lacunae around them were empty. No osteoblasts were observed at the border (Figure 6 and Figure 7).

**Figure 8:**
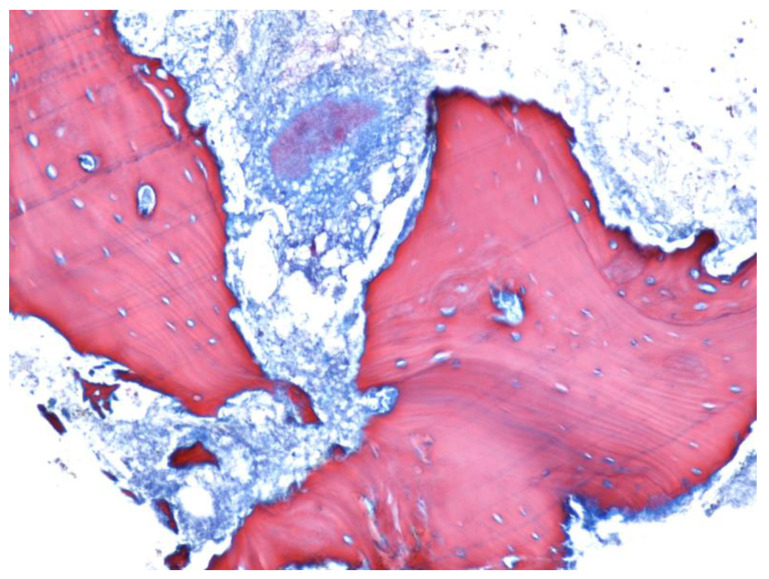
Osteonecrosis area, with Actinomyces colonies (Trichrome staining, ×100).

On the trichromic staining histopathological image, the bacterial colonies appeared as areas with a characteristic radial disposition in the center of the area with necrotic, acellular bone trabeculae, without having reactive bone (Figure 8).

**Figure 9:**
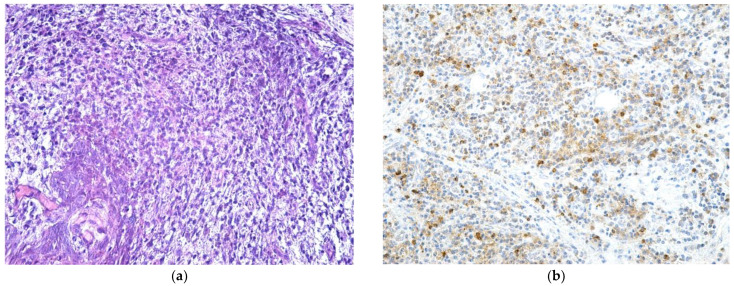
(**a**) Abundant inflammatory infiltrate in deep periodontium. Defense reaction of the body around the area of necrosis with neutrophil, lymphocyte, and macrophage cells (HE staining, ×200); (**b**) CD3 positive T lymphocytes are more numerous than B lymphocytes but more diffused distributed (CD3, ×200).

In the specimens with maxillary osteonecrosis, the deep marginal periodontium around the necrosis area had, as a defense reaction of the body, an abundant inflammatory infiltrate with neutrophils, lymphocytes, and macrophages. From the entire study group, 47 patients (representing 92.16%) had inflammatory infiltrate (Figure 9a,b).

The remaining four patients had ages above 66 years old and showed an equal gender distribution, three of them were from Constanța, and all had posterior mandibular MRONJ. None of these four patients presented bacterial colonies. In fact, there was a statistically significant association between the presence of inflammatory infiltrate and bacterial colonies, as assessed by Fisher’s exact test, *p* = 0.043. Further analysis indicated no significant association with other acquired parameters.

**Figure 10:**
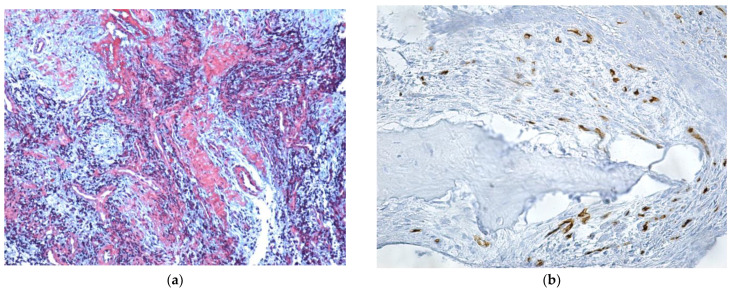
(**a**) Diffuse inflammatory infiltrate that alters (transects) the structure, chronic abundant inflammatory infiltrate of lympho-plasmocytic type, and numerous blood capillaries with turgid endothelium. Blood vessels and collagen fibers (in red) (HE staining, ×100); (**b**) perivascular B lymphocytes in foci of bone osteonecrosis (CD20, ×200).

Approximately three quarters of the patients presented lymphoplasmacytic infiltrate (Figure 10a,b) (39 patients, representing 76.47%), mostly females, but no significant association with gender was identified. The only statistically significant association was identified with the presence of viable bone in the evaluated sample (Fisher’s exact test, *p* = 0.047).

**Figure 11:**
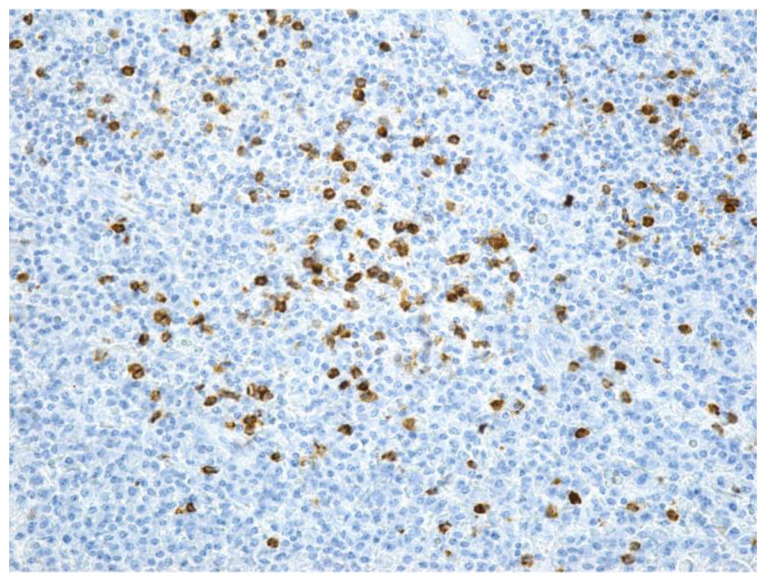
Numerous macrophages marked with CD68—inflammatory infiltrate in periodontium (CD68, ×200).

Along the junction between the periodontium and the area of bone necrosis, numerous macrophages were noticed. Macrophages (Figure 11) were only found in the histopathological examinations of three patients (5.88%), two females and one male, all with posterior MRONJ and ages older than 82 years old. There was a statistically significant association between the presence of macrophages and advanced age (above 75 years old), as assessed by Fisher’s exact test, *p* = 0.027.

**Figure 12:**
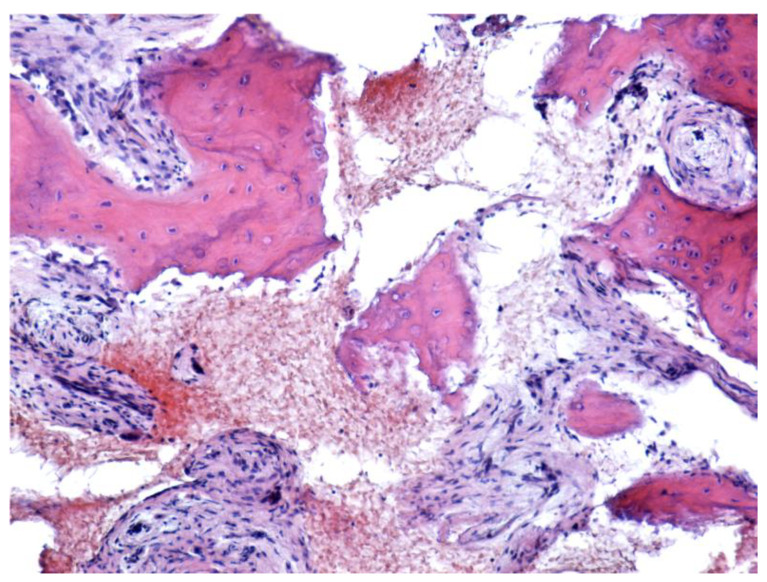
Large osteonecrosis field with fragmented bone and no osteoblasts. Reactive conjunctive tissue delimiting the necrotic tissue, surrounded by bone tissue with osteocytes-viable bone (HE staining, ×100).

Histopathological exam revealed the existence of viable bone (Figure 12) for 16 patients (31.37%), mostly from Craiova (11 patients). Half of these patients also developed bacterial colonies. There was an almost equal distribution between maxillary and mandible localization. As mentioned above, a lymphoplasmacytic infiltrate was found in nearly 94% of patients with viable bone (*p* = 0.047).

## 4. Discussion

MRONJ occurs in patients with breast and prostate cancer treated with bisphosphonates [23,24], especially zoledronic acid [24] and with other medications for bone metastases, such as denosumab [15,25]. In the reviewed literature, compared to other bisphosphonates, zoledronic acid is responsible for most of MRONJ-type adverse effects in these patients [11]. The patients with MRONJ included in this study had previously been treated monthly with zoledronic acid 4mg intravenously [25,26] for bone metastases associated with breast, prostate, and other forms of cancer (cervical, lung, colon, and multiple myeloma). Zoledronic acid is 100 up to 1000 times more effective than pamidronate [27], it is injected intravenously, and the dose is substantial in comparison to what is given for osteoporosis [28,29].

Studies have shown that patients receiving bisphosphonates intravenously have an increased risk of MRONJ compared to patients receiving oral bisphosphonates [16,17]. Moreover, the onset time of MRONJ is faster in patients receiving intravenous bisphosphonates than in those receiving oral bisphosphonates [16]. Awareness of patients treated with bisphosphonates, as well as increasing the level of their health education, have an important role in preventing the occurrence of osteonecrosis [30].

Inflammatory and circulatory transformations occur with age, as do immune disorders, making women aged 50 to 60 more prone to developing MRONJ compared to men of the same age [31]. Menopausal female patients treated with nitrogen-containing bisphosphonates are at a “10-fold higher risk to develop MRONJ at age 55 or older compared to young female patients” [14,32]. Another two studies highlighted the fact that the incidence of cases of bisphosphonate-related osteonecrosis of the jaw has been increasing in the recent years and showed that, at a mean age of 62, there were more women affected than men [33,34]. In the current study too, women aged 60–70 developed MRONJ in a higher percentage compared to men in the same age group.

Most patients in the current study had MRONJ in advanced stages (2 or 3), complaining of pain, erythema, purulent discharge, and extraoral fistulas/oroantral communication [35]. The lack of dental surveillance for these individuals led to the diagnosis of MRONJ at advanced stages. MRONJ was triggered by alveolar surgery (dental extractions) and periodontal and periapical inflammatory disease [36]. According to the Italian Consensus 2020 and AAOMS 2022 recommendations, cancer patients receiving bisphosphonates should have a dental exam before beginning MRONJ administration [35,37] and should be monitored during therapy [38]. By control strategies for conditions such as periodontal disease or periapical inflammation [35], this strategy would reduce the overall number of patients with adverse effects, such as MRONJ [39,40].

Extraction was a triggering factor for a large number of the cases, but periapical disease and periodontal disease were also encountered [9,41]. These two inflammatory conditions could also be a cause for dental extraction, along with vertical root fracture. Extraction could occur if either the patients did not disclose the necessary data in the anamnesis to the dentist who performed the extraction in order for the dentist to take the necessary precautions to prevent MRONJ, or if MRONJ was already installed in stage 1, allowing the evolution of MRONJ to continue after the extraction [42]. According to several studies, the prevalence of estimated MRONJ occurring following tooth extraction in cancer patients receiving IV bisphosphonates varies from 1.6% to 14.8% [14,19]. Avishai G et al. showed that the presence of local inflammation or infection facilitated the installation of MRONJ in patients having tooth extractions, increasing the risk by more than ten-fold [9]. An alternative to extraction would be to maintain the irretrievable teeth on the arch once the prosthetic work is removed and the roots are endodontically treated [14].

Localization of MRONJ was in the mandible in over two-thirds of cases, the mandible being a less vascularized bone [43,44]. AlRowis et al. found that the occurrence of MRONJ was more common in the mandible (73%) than in the maxilla (22.5%), in a review published in 2022 [18]. Similarly, in a study performed on CT scans of patients with MRONJ, Baba et al. showed that 78.7% had MRONJ in the mandible. The anterior or posterior placement of MRONJ in the jaws (mandible or upper jaw) was debated and MRONJ was found to be located in the posterior area in 84% of cases [45]. Other authors also showed that in few cases, MRONJ could occur simultaneously in both jaws (4.5%) [46,47]. In a randomized study, Jeong et al. reported a frequency of only 0.82% of MRONJ after maxillary tooth extractions compared to a frequency of 5.24% after mandibular tooth extractions, registering a statistically significant difference [48]. Also, the posterior area of the jaws was more frequently affected by MRONJ than the anterior area [43,49].

Marx explained this phenomenon, reporting that the incidence of MRONJ in the oral cavity is higher in areas with high mechanical loading on the bone (posterior mandibular lingual cortex, edentate alveolar ridge, and hard lamina). Mechanical stress of the bone causes decreased osteocyte OPG (osteoprotegerin) secretion, increased RankL/OPG ratio, and increased recruitment of osteoclasts for resorption of the bone [50]. In certain regions, osteocytes detect mechanical strain on the bone and send bone resorption signals [50,51,52]. Excessive or insufficient mechanical pressure in the maxillary bone (as it is with edentate ridge overloading under prostheses, occlusal trauma in periodontal disease, as well as the absence of mechanical periapical bone in root residues, etc.) triggers osteocyte apoptosis [51,52,53]. Although the apoptosis of osteocytes is essential for replacing the damaged bone [54], in the aged bone it does not take place normally, so bone homeostasis is regulated through the senescent osteocytes that increase their number of dendritic processes [32,55]. If the bone contains nitrogen-containing bisphosphonates, due to the apoptosis of the osteoclasts (bone cells that initiate the remodeling of the bone), the bone no longer regenerates its osteocytes and remains with empty lacunae.

Once the patients came to the maxillofacial surgeon, MRONJ evaluation was carried out with CT examination, as recommended by the Italian Consensus 2020 [37]. On CT images, MRONJ appears as radiolucent areas of osteolysis, sometimes with areas of bone sequestrum surrounded by osteolysis.

Scientific societies that recommend MRONJ management, such as AAOMS [8], the Italian Society of Maxillofacial Surgery (SICMF), and the Italian Society of Pathology and Oral Medicine (SIPMO) [37], agree that minimally invasive treatment, with systemic broad-spectrum antibiotics and topical antiseptics is the best. According to Pardo-Zamora et al. [35], the best antibiotic schedule for the treatment of osteonecrosis is not well defined.

Marx [29] recommended penicillin derivatives as first choice drugs. The antibiotic prescribed should cover *Actinomyces*, *Fusobacterium*, *Eikenella*, *Bacillus*, *Staphylococcus*, *Streptococcus*, and *Treponemas* [35].

Whenever feasible, an antibiogram should be conducted from the patient’s first presentation to the oral surgeon; however, if this is not possible, beta-lactams (amoxicillin or amoxicillin + clavulanic acid) are the most suitable antibiotics available for non-allergic individuals [22,35]. Clindamycin should be a potential antibiotic for allergic patients [37], but *Staphylococcus aureus* and *Pseudomonas mendocina* may be resistant, so the available antibiotics should be ceftazidime, ceftriaxone, and ciprofloxacin, as our results revealed.

As recommended by the Italian Consensus 2020, surgical treatment is performed early whenever indicated to reduce the surgical trauma of patients with MRONJ and increase long-term healing [37]. Since in the early years after the discovery of this adverse effect, cases treated with bone resection were much more numerous [49,56] due to the serious consequences of resection and the fact that rehabilitation is much more difficult, this type of treatment was gradually abandoned and minimally invasive surgery was chosen in most cases, as occurred in the current study. As a result, instead of maxillary bone resections, sequestrectomy was performed together with curettage of the residual bone until clear bleeding occurred. To prevent further bone exposure, the remaining bone surfaces were beveled [19,37,57].

The surgical treatment was planned individually depending on the patient’s general state of health and the impact of the surgical intervention [37]. The therapy with bisphosphonates in the patients included in this study was continued in most cases because they already had long-term treatment established, on average over 2 years, and the half-life of the bisphosphonates administered intravenously was very long. Several international forums (AAOMS, Italian consensus, etc.) have recommended that in cancer patients under intravenously therapy with bisphosphonates, the administration of the bisphosphonates should not be interrupted in case of surgical treatment [19,35,57,58].

Platelet-rich fibrin (PRF) could be used as a complementary therapy for improved healing [59,60]. PRF membranes and clots can be employed to enhance the results of surgical therapy of recurrent MRONJ, with excellent results in treating stage 2 of this condition. Unfortunately, this therapy was only available to a limited number of patients.

Histopathological analysis of collected bone fragments was conducted on all patients in the research, in accordance with the Italian consensus 2020 [37]. Since bisphosphonates begin the activity at the level of the Haversian canals (vascular source), the bone tissue tries to seize the necrosis area that commences at the level of these canals. Necrosis of the bone begins with demineralization, the death of osteocyte cells in their lacunae, the death of osteoblasts and osteoclasts, and the destruction of the bone progressing to the stage of total necrosis. In the study of Bedogni et al. also, the specimens obtained from the areas of osteonecrosis had a large part of nonvital bone, with irregular edges and empty osteocyte lacunae. Osteoblasts and osteoclasts were almost absent and blood vessels were rare, without signs of bone remodeling [61].

The appearance of advanced osteonecrosis sections was inhomogeneous, with uneven margins and a vacuolar, moth-eaten appearance. This argument has been highlighted in both this study and other research, such as those conducted by Lu and Petia [62,63].

In the current study, necrosis regions alternated with areas of bone tissue in various phases of deterioration, ranging from intact bone tissue or bone tissue with incipient osteonecrosis to totally necrotic bone tissue. Paparella et al. also noticed the mosaic appearance of the bone [64]. The authors discovered that MRONJ had histological features similar to osteomyelitis, such as bone necrosis and bone marrow containing cellular detritus, bacterial colonies, and an inflammatory infiltrate. All trabeculae present in the specimens were necrotic and thicker than usual (bone sclerosis) and had a Paget’s disease-like appearance with obvious signs of remodeling. These characteristics resulted in a mosaic pattern with a multi-compartment configuration. Most of these compartments had no obvious connection to the bone marrow. As a result, these bone areas were not connected to the source of nutrition, compromising tissue vitality and response to harmful factors (bacteria and trauma), facilitating the development of osteonecrosis [64].

Unlike the current investigation, Favia et al. found several osteoclast-type cells adjacent to the interface of the residual bone spicules in the necrotic regions. Osteoclastic cells were small and had 3–5 nuclei. These areas were filled with an inflammatory infiltrate composed of polymorphonuclear phagocytes, plasma cells, monocytes, lymphocytes, acellular necrotic detritus, thin-walled dilated blood vessels, and residual and intensely basophilic bone spicules. They had bone destruction and massive resorption with irregular margins (Howship lacunae). In addition, centrifugal bone resorption was seen in the Haversian canals, wider than those in non-necrotic locations and with an exceedingly uneven contour. However, in areas with necrosis, the bone structure was acellular and wide, with irregular Haversian canals and inflammatory infiltrate [65].

In our study, macrophages were observed on the HP images only in a few cases. This is due to the bisphosphonates reducing the macrophage activity, as demonstrated by Sabatino et al. [66]. Macrophages/monocytes have an important role in the immune defense system because they act as antigen-presenting cells. By affecting them, bisphosphonates suppress the immune response of the bone in patients with MRONJ, making it sensitive to bacterial colonies, thus explaining the presence of bacterial colonies on the HP images as well as the clinical signs of infection [67].

Adequate monitoring of patients receiving bisphosphonates can reduce the risk of osteonecrosis and prevent complications [68].

## 5. Conclusions

MRONJ occurring in cancer patients treated with zoledronic acid is an important complication that greatly reduces the quality of life and survival of these patients. These patients are not usually monitored by the dentist and, as a result, they are discovered in advanced stages of MRONJ. MRONJ was detected in the studied groups mainly in the mandible’s posterior regions. The trigger factor was, in most cases, tooth extraction but also periapical or periodontal infections. In most cases, the surgical treatment was carried out by curettage and sequestrectomy, with beveling of the sharp bone edges and covering the wound with a muco-periosteal flap. Histopathological examination of the fragments collected after the surgical treatment revealed osteonecrosis specific aspects, including the lack of bone cells, inflammatory infiltrate, and bacterial colonies. The risk of osteonecrosis as well as its complications can be controlled by carefully monitoring the patients treated with bisphosphonates.

## Figures and Tables

**Table 1:** Distribution of the study lot according to city and the main clinical parameters.

Parameter	Values	Craiova (no/%)			Constanța (no/%)			Total	*p* **
		Total (100%)	Females	Males	Total (100%)	Females	Males		
Number (%)	-	26	17 (65.38%)	9 (34.62%)	25 (100%)	15 (60%)	10 (40%)	51	-
Age	<70 *	12	6 (50%)	6 (50%)	13	8 (61.54%)	5 (38.46%)	25	0.676
	≥70 *	14	11 (78.57%)	3 (21.43%)	12	7 (58.33%)	5 (41.67%)	26	
Age (mean ± SD)	<70 *	61.50 ± 5.80	62.66 ± 3.72	60.33 ± 7.55	64.49 ± 4.19	63 ± 4.56	67.4 ± 1.14	-	-
	≥70 *	76.35 ± 4.21	75.81 ± 4.33	78.33 ± 3.78	78.66 ± 4.73	79.4 ± 4.31	77.6 ± 5.59	-	
Localization	Mandible	19	12 (63.16%)	7 (36.84%)	15	8 (53.33%)	7 (46.67%)	34	0.322
	Maxilla	7	5 (71.43%)	2 (28.57%)	10	7 (70%)	3 (30%)	17	
Position in dental arch	Posterior	24	16 (66.67%)	8 (33.33%)	24	14 (58.33%)	10 (41.67%)	48	1.00 ***
	Anterior	2	1 (50%)	1 (50%)	1	1 (100%)	0 (0%)	3	
Stage	2	21	13 (65%)	7 (35%)	16	13 (65%)	7 (35%)	37	0.180
	3	5	4 (66.67%)	2 (33.33%)	9	2 (40%)	3 (60%)	14	
Intervention	Sequestrectomy	22	13 (86.67%)	2 (13.33%)	24	15 (65.22%)	8 (34.78%)	46	0.350 ***
	Resection	4	4 (36.36%)	7 (63.64%)	1	0 (0%)	2 (100%)	5	
	No	15	9 (60%)	6 (40%)	20	10 (50%)	10 (50%)	35	

* Years old. ** Chi-square test. *** Fisher’s exact test.

**Table 2:** Distribution of the study lot according to city and the main studied parameters.

Parameter	Values	Craiova (no/%)			Constanța (no/%)			Total	*p* *
		Total (100%)	Females	Males	Total (100%)	Females	Males		
Bacterial colonies	Yes	20	14 (70%)	6 (30%)	7	5 (71.43%)	2 (28.57%)	27	<0.0005
	No	6	3 (50%)	3 (50%)	18	10 (55.56%)	8 (44.44%)	24	
Epithelium	Yes	7	6 (85.71%)	1 (14.29%)	16	9 (56.25%)	7 (43.75%)	23	0.008
	No	19	11 (57.89%)	8 (42.11%)	9	5 (55.56%)	4 (44.44%)	28	
Fibrous tissue	Yes	7	8 (80%)	2 (20%)	19	13 (72.22%)	5 (27.78%)	26	<0.0005
	No	19	9 (56.25%)	7 (43.75%)	6	2 (28.57%)	5 (71.43%)	25	
Granulation tissue	Yes	12	10 (76.92%)	3 (23.08%)	18	11 (61.11%)	7 (38.89%)	30	0.061
	No	14	7 (53.85%)	6 (46.15%)	7	4 (57.14%)	3 (42.86%)	21	0.061
Inflammatory infiltrate	Yes	25	16 (64%)	9 (36%)	22	14 (63.64%)	8 (36.36%)	47	0.350 **
	No	1	1 (100%)	0 (0%)	3	1 (33.33%)	2 (66.67%)	4	
Lymphoplasmacytic infiltrate	Yes	19	13 (68.42%)	6 (31.58%)	20	14 (70%)	6 (30%)	39	0.560
	No	7	4 (57.14%)	3 (42.86%)	5	1 (20%)	4 (80%)	12	
Macrophages	Yes	2	2 (100%)	0 (0%)	1	0 (0%)	1 (100%)	3	1.00
	No	24	15 (62.50%)	9 (37.50%)	24	15 (62.50%)	9 (37.50%)	48	
Hemorrhagic infiltration	Yes	11	9 (64.29%)	5 (35.71%)	12	8 (72.73%)	3 (27.27%)	23	0.683
	No	15	8 (66.67%)	4 (33.33%)	13	7 (50%)	7 (50%)	28	
Viable bone	Yes	11	8 (72.73%)	3 (27.27%)	5	5 (100%)	0 (0%)	16	0.086
	No	15	9 (60%)	6 (40%)	20	10 (50%)	10 (50%)	35	

* Chi-square test. ** Fisher’s exact test.

## Data Availability

The authors declare that the data of this research are available from the corresponding authors upon reasonable request.
